# Clinical characteristics of hepatic Arterioportal shunts associated with hepatocellular carcinoma

**DOI:** 10.1186/s12876-018-0899-3

**Published:** 2018-11-12

**Authors:** Huiyong Wu, Wei Zhao, Jianbo Zhang, Jianjun Han, Shuguang Liu

**Affiliations:** 1 Department of Intervention, Shandong Tumor Hospital Affiliated to Shandong University, Jinan, 250117 China; 2 Department of Radiotherapy, Shandong Tumor Hospital Affiliated to Shandong University, Jinan, 250117 China; 3 Department of pathology, Shandong Tumor Hospital Affiliated to Shandong University, Jinan, 250117 China; 4 Department of Thoracic Oncology Surgery, Shandong Tumor Hospital Affiliated to Shandong University, No. 440, Jiyan Road, Jinan, 250117 China

**Keywords:** Hepatocellular carcinoma, Hepatic arterioportal shunts, Portal vein tumor embolus, Transarterial chemoembolization

## Abstract

**Background:**

Hepatic arterioportal shunt (A-P shunt) is defined as the direct blood flow established between hepatic artery and portal venous system; it is frequently observed in patients with hepatocellular carcinoma (HCC). Clinically, it is important to diagnose HCC associated A-P shunts, as it may impact the treatment strategy of the patients. In the present study, we described the imaging findings of the HCC associated A-P shunts and discussed the treatments strategy of such patients. From the findings, we also discussed the potential cause of A-P shunts.

**Methods:**

Clinical data of HCC patients (*n* = 560), admitted to the hospital between April 2012 to April 2014, were reviewed. Hepatic angiography was used to examine the presence of A-P shunts. Of the 137 patients with A-P shunts, grading of the A-P shunts was performed, and statistical analysis of the different grades of A-P shunts and clinical characteristics was performed.

**Results:**

The hepatic angiography confirmed that 99 patients had typical A-P shunts (Grade 1–3), and 38 patients had atypical A-P shunts. Embolization was the main strategy used to treat A-P shunts, in which liquid embolic agents appeared to provide a better treatment outcome. The correlation analysis showed that the grading of portal vein tumor thrombus was significantly associated with the grading of A-P shunt (*p* = < 0.001, Spearman correlation coefficient was 0.816 ± 0.043).

**Conclusions:**

We characterized A-P shunts and proposed treatment strategy for treating HCC patients with various levels of A-P shunts. The findings supported the hypothesis that the formation of HCC associated A-P shunts was caused by tumor thrombus.

## Background

Transcatheter arterial chemoembolization (TACE) is one of the important treatment strategies for patients with hepatocellular carcinoma (HCC). It is not only used as a standard treatment for middle-stage tumor but also as a necessary treatment for early stage patients after surgery [1, 2]. Hepatic arterioportal shunts (A-P shunts) are frequently observed in patients with HCC. The presence of A-P shunt often complicates the HCC cases and severely affects the efficacy and safety of TACE. For example, the A-P shunts may cause the chemotherapy drug and embolic agent to run-off through the shunt path. The hepatic A-P shunts may also lead to the tumor thrombus detached from hepatic artery, resulting in a blockage of portal vein. The tumor thrombus detached may also block the hepatic small arteries, leading to hepatic tissue necrosis [3–6]. As such, the best treatment strategy for HCC complicated with the hepatic A-P shunts remains to be determined.

There are various causes of A-P shunts, such as liver cirrhosis, hepatic neoplasms, hepatic trauma, obstruction of the portal or hepatic vein, and inflammatory diseases [7]. For HCC patients, it was suggested that A-P shunts were caused by the invasion of HCC into the portal vein system. When the hepatic vein is obstructed (e.g. due to rumor thrombus), the pressure gradient between the sinusoids and portal veins is reversed, resulting in a functional A-P shunt [8, 9]. The hemodynamics in such cases seem to be very complex and remain to be fully investigated. Histological findings have shown that when tumor tissues invaded along the portal vein cavity, the portal vein structure was generally complete, and structural changes of the portal vein have rarely been observed. On the basis of the hepatic artery angiography findings, direct circulation between hepatic artery and portal vein was not found in cases of tumor invasion. We hypothesized that hepatic A-P shunts of HCC patients were caused by the portal vein tumor thrombus. In this study, we analyzed characteristics of hepatic artery angiography images of A-P shunts in 137 HCC patients, and described the treatment strategy for such patients. We also performed statistical analysis to investigate the correlation of portal vein tumor thrombus and A-P shunts.

## Patients and methods

### Patients

Clinical data of 560 HCC patients, admitted to Shandong Tumor Hospital Affiliated to Shandong University between April 2012 and April 2014, were reviewed. All of these patients received TACE or transcatheter arterial embolization (TAE) treatment. Among these patients, 137 of them (124 male and 13 female) had A-P shunts. The age of patients with A-P shunts ranged from 32 to 83 years old, with median at 55 years old. The eligibility criteria include: 1) confirmed diagnosis of HCC by biopsy or imaging according to accepted guideline [10]; 2) observation of hepatic A-P shunt in hepatic arteriography; 3) if A-P shunt was not found on hepatic arteriography, observation of iodized oil flowing from hepatic artery to the portal vein must be observed during TAE under X-ray examination; 4) patients with no history of hepatic resection. In this cohort, 127 patients had history of hepatitis B virus infection, 2 patients had hepatitis C virus infection, and the rest had no liver disease of clinical significance. Table 1 summarized the baseline clinical characteristics of the patients. The A-P shunts were divided into four grades: the atypical A-P shunts were classified as Grade 0, while the typical A-P shunts were further classified into three grades (Grade 1–3) according to severity (Table 2).

**Table 1:** Characteristics of patients with typical and atypical A-P shunts

Characteristics	Number of patients or Mean/Median	Atypical A-P Shunt	Typical A-P Shunt	*p*
Sex, *n* (%)				0.945
Male	124 (90.51)	35 (92.11)	89 (89.90)	
Female	13 (9.49)	3 (7.89)	10 (10.10)	
Age, mean ± sd	54.91 ± 9.47	56.45 ± 8.0	54.32 ± 9.94	0.241
Etiology, *n* (%)				0.837
HBV	127 (92.70)	35 (92.11)	92 (92.93)	
HCV	2 (1.46)	1 (2.63)	1 (1.01)	
Others	8 (5.84)	2 (5.26)	6 (6.06)	
Number of tumors, *n* (%)				0.055
Multiple	86 (62.77)	19 (50.00)	67 (67.68)	
Single	51 (37.23)	19 (50.00)	32 (32.32)	
Child-Pugh score, *n* (%)				0.898
A	102 (74.45)	28 (73.68)	74 (74.75)	
B	35 (25.55)	10 (26.32)	25 (25.25)	
AFP(ng/ml), median (quartile)	309.0(13.85, 1210.0)	57.0 (7.2,1210.0)	579 (15.7,1210.0)	**0.035**
Albumin(g/L), mean ± sd	40.11 ± 5.34	39.59 ± 5.71	40.32 ± 5.21	0.827
Bilirubin (mmol/L), median (quartile)	22.0 (15.5,29.6)	20.95 (14.3,28.5)	22.2 (15.5,30)	0.644
Creatinine (umol/L),median (quartile)	62.0 (54.2, 71.0)	60.65 (53.7,68)	63.1 (54.2,73.1)	0.701
AST(U/L),median(quartile)	41.0 (28.0, 65.0)	32.05 (26.4,58.5)	43.3 (30.0,67.0)	**0.025**
ALT(U/L),median(quartile)	49.0 (36.0, 74.9)	39.5 (29.3, 74.0)	53 (37.8, 77.0)	0.106
Platelets (10^9^/L) median(quartile)	125 (88, 282)	109 (91, 161)	128 (87, 182)	0.341
Tumor size (cm), median (quartile)	6.5 (3.8, 9.5)	5 (3.3, 7.0)	7.1 (4.5, 10.5)	**0.007**

**Table 2:** Grading of A-P shunt

Grade	Definition	Number of patients
0	A-P shunt was not observed in hepatic arterial angiography. The iodized oil was dispersed from hepatic artery into the portal vein, leading to the deposition of iodized oil in intrahepatic portal vein unrelated to tumor.	38
1	Segmental branches of the portal vein portal was observed due to A-P shunt	21
2	Portal vein trunk was observed due to A-P shunt located in the same hepatic lobe	47
3	Portal vein trunk was observed due to A-P shunt located in a different hepatic lobe	31

### Treatment with TACE/TAE

Digital subtraction angiography was performed on hepatic artery or superior mesenteric artery in all patients. Iodized oil (5–20 mL) and doxorubicin (40–60 mg) were used in arterial embolization. According to the timing of visualization of the venous structures on imagining, embolic agents including microsphere, absolute ethanol, and coils were used for shunt embolization. After embolization, hepatic artery angiography and liver-enhanced CT/MR were performed to examine the portal vein, specifically to observe the presence of tumor thrombus in the main trunk or branches of the portal vein.

### Statistical analysis

Categorical variables were presented in frequency (%), and continuous variables were presented in mean ± standard deviation, or median. Categorical data between two groups were compared using the chi-square test or Fisher exact test. Continuous data between two groups were compared using t-test if the data were normally distributed; otherwise, Wilcoxon two-sample test was used. The association of the grading of A-P shunt and the grading of portal vein tumor thrombus was analyzed using Spearman rank correlation analysis. All statistical analyses were performed using SAS9.3. Two-tailed testing was used, and *p* < 0.05 was defined as statistically significant.

## Results

### Imaging and clinical characteristics of typical and atypical A-P shunt

**Fig. 1:**
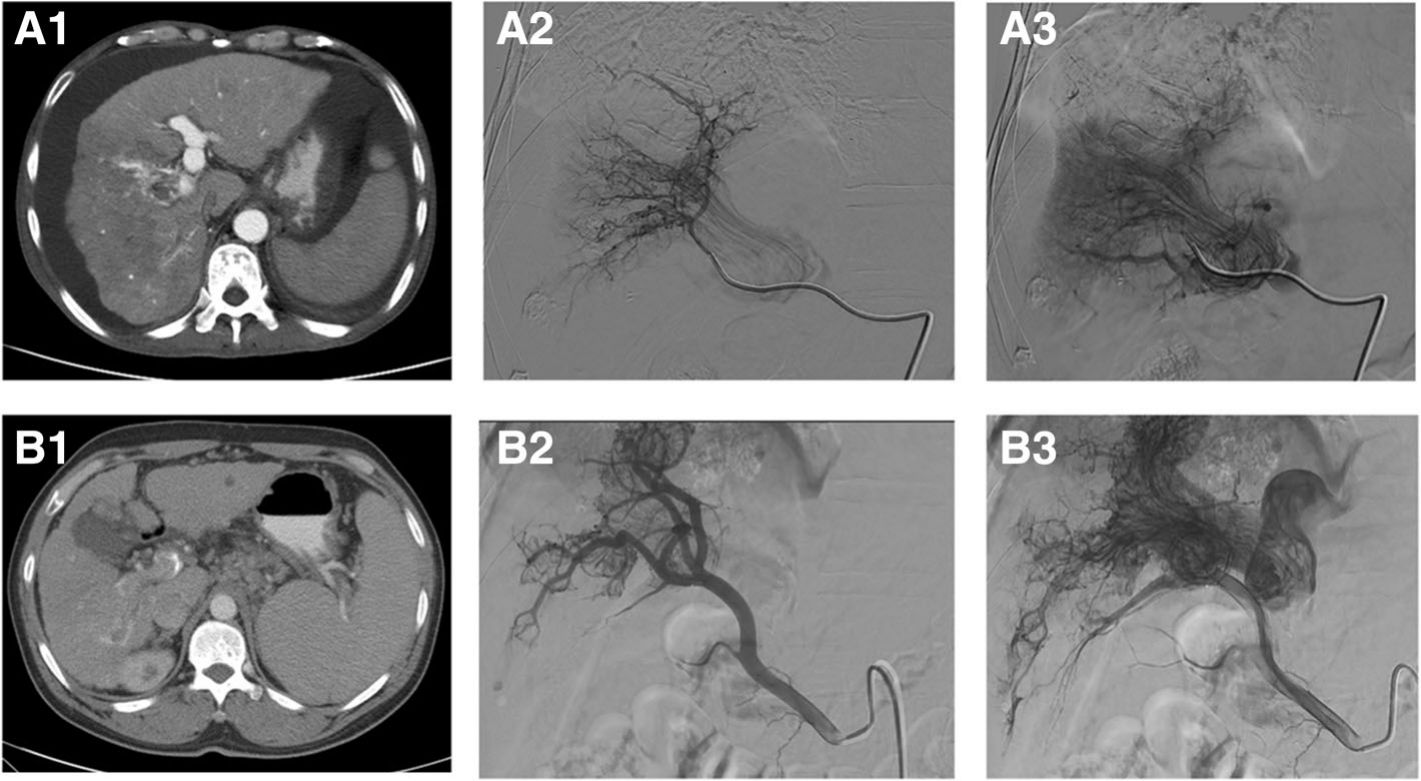
**A**. A patient with tumor thrombus and A-P shunt. (A1) Liver CT image showed the presence of right portal vein tumor thrombus. Arteries around the tumor thrombus were filled with contrast agent, suggesting the presence of A-P shunt. The intra-luminal filling of embolus indicated the internal blood flow. (A2) Early phase hepatic angiography showed ‘thread and streaks’ signs, corresponding to the blood spaces and vessels, which run longitudinally in and around the tumor thrombus. (A3) Late phase hepatic angiography showed that the blood flow of the tumor thrombus reached the portal vein, leading to the enhancement of portal vein. **B**. CT images of another patient with tumor thrombus and A-P shunt. (B1) CT image showed tumor thrombus in the right portal vein branch. Staining of the arteries around thrombus suggested the presence of A-P shunt. (B2) Early phase hepatic angiography showed the hepatic arteries run longitudinally in the hepatic portal vein. (B3) Late phase hepatic angiography: blood vessels in the embolus showed ‘tread and streaks’ signs. Enhancement of the left branch of the portal vein and segment of portal vein proximal to the embolus

A-P shunts were classified into two types (typical and atypical) according to the hepatic arteriogram images. In typical A-P shunt, early enhancement of portal vein was observed (Fig. 1). The detail of the blood flow of the A-P shunts was displayed clearly, with the images showing ‘thread and streaks’ signs, corresponding to the blood flow and vessels. According to the conditions of the blood supply of tumor and the severity of A-P shunts, embolic agents including microsphere, absolute ethanol, and spring coils were used for shunt embolization.

In atypical A-P shunt, portal vein was not enhanced during the hepatic arteriogram examination. In these patients, when iodized oil was injected, the flow of oil from portal artery through the A-P shunt and through the portal vein was observed. Also, the short-term retention of iodized oil in the portal vein and diffused distribution of iodized oil in the liver were observed. This observation was different from the over-embolization of iodized oil, in which the iodized oil was first accumulated in the tumor and then in the peripheral portal vein. Of the 137 patients, 99 of them had typical A-P shunts and 38 had atypical A-P shunts. The comparison the clinical characteristics of the two groups of patients showed that patients with typical A-P shunts had significantly higher levels of serum AFP (*p* = 0.035) and AST (*p* = 0.025). Also, they had significantly larger tumor (*p* = 0.007), suggesting the advanced HCC stage of these typical A-P shunt patients (Table 1).

### Grading of A-P shunts and portal vein tumor thrombus

**Fig. 2:**
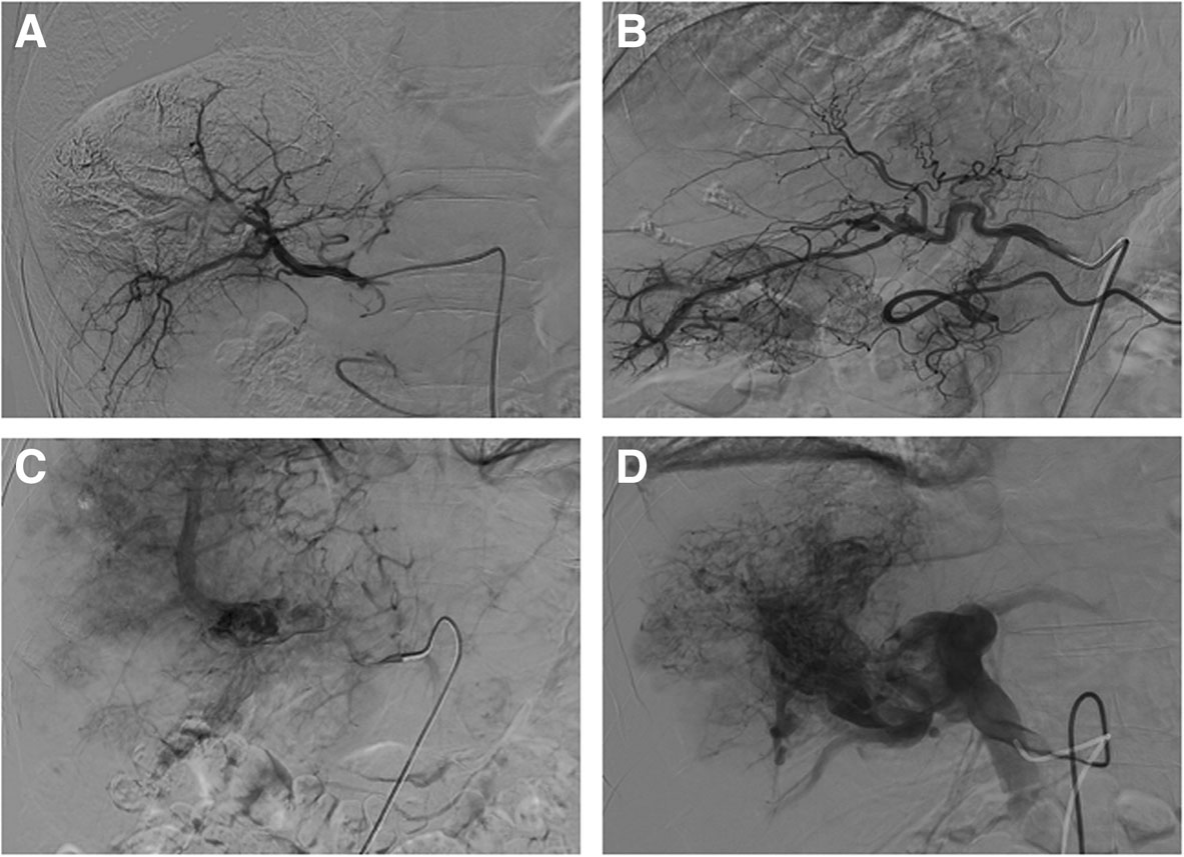
Representative images of hepatic arteriogram showing different grades of A-P shunts. **a** Grade 0: A-P shunt was not observed. **b** Grade 1: enhancement of the VI segment of the portal vein in the right lobe of the liver. **c** Grade 2: enhancement of the right portal vein and portal vein tumor thrombus. **d** Grade 3: enhancement of right portal vein main branches and trunk, with ill-defined intra-luminal filling defect. A large number of disorganized arteries providing blood supply for the portal vein thrombus was observed

We further classified the A-P shunts into four grades (Table 2). For the 99 cases of typical A-P shunts, 21 of them were Grade 1, 47 patients were Grade 2, and 31 patients were Grade 3 (Fig. 2). We also examined the presence of portal vein tumor thrombus in all patients, and found 85.4% (117/137) of them had tumor thrombus. The tumor thrombus was classified into three grades (Grade 1–3) according to the severity (Table 3). There were 34 patients with Grade 1 tumor thrombus, 50 patients with Grade 2, and 33 patients with Grade 3.

**Table 3:** Grading of portal vein tumor thrombus

Grade	Definition	Number of patients
0	No tumor thrombus. Only the portal venous invasion was observed.	20
1	Tumor thrombus was observed in branches of portal vein.	34
2	Tumor thrombus was observed in the right or left branches of portal vein.	50
3	Tumor thrombus was observed in the portal vein trunk.	33

### Association of A-P shunt and portal vein tumor thrombus

To investigate if the grading of A-P shunts was associated with the grading of tumor thrombus, a correlation analysis was performed. The grading of portal vein tumor thrombus was significantly associated with the grading of A-P shunt (*p* = < 0.001, Spearman correlation coefficient was 0.816 ± 0.043) (Table 4). For patients without thrombus, 19/20 of them had Grade 0 A-P shunt. There was only one patient with Grade 2 A-P shunts showed the absence of HCC invasion and absence of portal vein tumor thrombus. This patient had dilated bile duct as shown by the hepatic angiography.

**Table 4:** Correlations between severity of hepatic A-P shunt and portal vein tumor embolus

Portal Vein Tumor thrombus Grade	Hepatic A-P shunt grade				Total	*p*	Spearman correlation coefficient
	0	1	2	3			
0	19 (50.00)	0 (0.00)	1 (2.13)	0 (0.00)	20	< 0.001	0.816 ± 0.043
1	17 (44.74)	11 (52.38)	6 (12.77)	0 (0.00)	34		
2	0 (0.00)	10 (47.62)	35 (74.47)	5 (16.13)	50		
3	2 (5.26)	0 (0.00)	5 (10.64)	26(83.87)	33		
Total	38	21	47	31	137		

There were 20 patients with no tumor thrombus observed (Grade 0) during the initial imaging examination. However, in the subsequent visits, 18 of these patients had tumor thrombus developed and typical A-P shunts observed. For the 34 patients, who had Grade 1 tumor thrombus, 17 of them had typical A-P shunts observed during the first imaging examination. Along the disease course of HCC and tumor thrombus development, eventually 28/34 patients showed typical A-P shunts by the end of the study.

## Discussion

A-P shunts are frequently observed in patients with advanced HCC. In a previous study, hepatic angiogram examination revealed that among the 114 patients with HCC, 63.2% of them had various levels of A-P shunts [11]. A recent study also showed that among 596 patients with HCC, 27% of them had severe A-P shunts [12]. HCC associated A-P shunts may occur in different routes. Okuda et al. reported that shunts could occur 1) through a tumor thrombus in the portal branch, 2) in a retrograde direction via a peripheral tumor nodule, 3) through a small tumor invading or amputating an artery, or 4) through a tumor located near a major portal vein branch and supplied by a large artery [11]. In the present study, almost all the HCC associated A-P shunts were associated with the tumor thrombus.

Digital subtraction angiography has been the gold standard for diagnosis of HCC-associated A-P shunts, but the detail of the blood flow could sometime be difficult to observe using this technique. To date, with the advances in imaging technology, the presence of A-P shunts could easily be detected through hepatic arteriogram. In this study, the detail of the blood flow of the A-P shunts was clearly displayed by imaging examination in majority of the patients with typical A-P shunts (99/137). The imaging showed ‘thread and streaks’ signs, corresponding to the blood flow and vessels; thrombus located in a large branch and/or trunk of the portal vein was also observed [13]. An increased number and thickening of artery branches in proximity to the bile duct were often observed, indicating an increase of blood supply to the tumor thrombus. Portal vein thrombus is often caused by the HCC tumors that invade through the stroma. The blood supply to the tumor thrombus increased with the growth of the thrombus, leading to the formation of typical A-P shunts.

In this study, most of the patients with atypical A-P had early-stage tumor thrombus. The early-stage thrombus had a relatively small amount of blood supply, thus low-flow of A-P shunts. In some patients with atypical shunt, portal vein thrombus was not found during the initial diagnosis. These patients had the HCC located near the portal vein, with invasion at the portal vein stroma. This condition is similar to the early-stage tumor thrombus. Indeed, these patients eventually developed tumor thrombus in the course of the disease. Based on the observations, we suggested that majority of HCC associated A-P shunts are related to the development of portal vein tumor thrombus.

It has been suggested that conventional TACE was not effective for HCC patients with significant A-P shunts. Efforts have been made to treat these patients with embolization materials such as gelatin sponges or coils; however, these approaches could only provide short-term benefits [14–16].The embolization materials (e.g. gelatin sponges, coils) are large in size, and are difficult to pass through the shunt and reach the portal vein. It has been reported that the use of iodized oil for embolization was effective in some patients with A-P shunts [14, 15]. We believe it is due to the effective embolization of the blood vessels of tumor thrombus. In our experiences, the use of iodized oil was effective in patients with low or absence of A-P shunts. Retentions of iodized oil in tumor thrombus were often observed in these patients after conventional TACE. Moreover, these patients with low level of A-P shunt may have better liver function. For patients with significant A-P shunts, materials such as sponge particle may first be used to induce shunt occlusion, followed by injection of other embolization agents [17]. Alternatively, the catheter could be inserted into the arteries of the thrombus for injection of embolization agents.

It has been reported that multiple TACE treatments may lead to A-P shunts, thus it is recommended that chemoembolization should be abandoned if A-P shunts appear or the dose of embolic agents should be reduced [18]. In the present study, some of the HCC cases continued to progress after TACE; invasion of the tumor at the portal vein and development of tumor thrombus were found. These conditions lead to an increase of A-P shunt flow. In other cases, TACE treatment did not stop the growth of the existing tumor thrombus, resulting in an increase of A-P shunts. Therefore, embolization of the tumor thrombus should be performed if patients have satisfactory liver function. The embolization of the blood supply of tumor thrombus could reduce the flow of the A-P shunts.

## Conclusions

In conclusion, the findings of the present study supported the hypothesis that the formation of HCC associated A-P shunts was caused by tumor thrombus. The grading of A-P shunts was significantly associated with the grading of tumor thrombus. It is recommended to perform embolization of the tumor thrombus, so that the flow of the A-P shunts may be decreased.

## Abbreviations

A-P shunts: Arterioportal shunts
HCC: Hepatocellular carcinoma
TAE: Transcatheter arterial embolization
